# Elucidation of Dietary Polyphenolics as Potential Inhibitor of Microtubule Affinity Regulating Kinase 4: In silico and In vitro Studies

**DOI:** 10.1038/s41598-017-09941-4

**Published:** 2017-08-25

**Authors:** Parvez Khan, Shafikur Rahman, Aarfa Queen, Saaliqa Manzoor, Farha Naz, Gulam Mustafa Hasan, Suaib Luqman, Jihoe Kim, Asimul Islam, Faizan Ahmad, Md. Imtaiyaz Hassan

**Affiliations:** 1 Center for Interdisciplinary Research in Basic Sciences, Jamia Millia Islamia, Jamia Nagar, New Delhi, 110025 India; 2 Department of Medical Biotechnology, Yeungnam University, Gyeongsan, 712-749 South Korea; 3 Department of Biochemistry, College of Medicine, Prince Sattam Bin Abdulaziz University, Al-Kharj, Saudi Arabia; 4 CSIR-Central Institute of Medicinal and Aromatic Plants, Lucknow, 226015 India

## Abstract

Microtubule affinity regulating kinase 4 (MARK4) is a Ser/Thr kinase belonging to AMPK-like family, has recently become an important drug target against cancer and neurodegenerative disorders. In this study, we have evaluated different natural dietary polyphenolics including rutin, quercetin, ferulic acid, hesperidin, gallic acid and vanillin as MARK4 inhibitors. All compounds are primarily binds to the active site cavity of MARK4. In silico observations were further complemented by the fluorescence-binding studies and isothermal titration calorimetry (ITC) measurements. We found that rutin and vanillin bind to MARK4 with a reasonably high affinity. ATPase and tau-phosphorylation assay further suggesting that rutin and vanillin inhibit the enzyme activity of MARK4 to a great extent. Cell proliferation, ROS quantification and Annexin-V staining studies are clearly providing sufficient evidences for the apoptotic potential of rutin and vanillin. In conclusion, rutin and vanillin may be considered as potential inhibitors for MARK4 and further exploited to design novel therapeutic molecules against MARK4 associated diseases.

## Introduction

Protein kinases are the most abundant enzymes encoded by human genome^1^. Many of these kinases are being targeted for inhibition during drug design and discovery^2, 3^. MARK4, a Ser/Thr kinase belonging to AMPK-like family, has recently become an important drug target against neurodegenerative diseases, cancer, obesity and other related metabolic disorders^4–9^. MARK4 was first identified by their ability to phosphorylate tau and other related microtubule associated proteins (MAPs) at specific Ser sites in KXGS motifs in the microtubule binding repeats^10, 11^. It helps in regulating stability of microtubules. MARK4 is a mammalian homologue of *C. elegans* Par-1 and plays an indispensable role in asymmetric cell division and establishment of cell polarity^12^. It also regulate cell cycle, cell signalling, cellular polarization, neuronal migration and differentiation^8, 13^. MARK4 shows highest expression in brain, kidney and testes^10, 14^. Whenever its expression fluctuates in cell it creates a havoc in many signalling pathways like Akt, mTOR, Wnt and NF-κB, and leads to a myriad of diseases as mentioned above^5, 15^. Recently, MARK4 has been reported to promote breast cancer cell proliferation and migration through the inhibition of hippo signalling^4^. Therefore, MARK4 is considered as an important target for design of drugs with anti-cancerous, anti-inflammatory and anti-neurodegenerative effects^6, 16–19^.

From the ancient time natural compounds or phytonutrients are known for their potential therapeutic applications and almost 60% of the drugs used in treating cancer are basically plant-derived compounds^20^. One of such class of compound is natural polyphenols like flavonoids, which are widely distributed in plants and generally present in food like herbs, nuts, vegetables, fruits, plant derived beverages like tea, coffee and red wine^21–24^. At present, a large number of flavonoids and its derivatives have been tested for their therapeutic properties^25–28^. Many epidemiological studies have shown that intake of polyphenols such as flavonoids reduce the risk of tumor, diabetes and neurodegeneration. As these phenolic compounds possess anticancer, antioxidant and anti-inflammatory activities, henceforth, the dietary polyphenols and flavonoids have gained a lot of attention in drug discovery^29, 30^. Furthermore, many studies and meta-analyses suggesting that there exist an inverse relationship between the consumption of flavonoids rich diet and development of many age-related disorders^31–33^.

Alzheimer’s disease (AD) is the most prominent example of neurodegeneration, effecting elderly population on large scale^34, 35^. Large number of reports are available on AD animal models suggesting that the dietary flavonoids act as a neuroprotective agent^36–40^. However, at present no explanation has been given to justify the association between consumption of flavonoids and better neurological health. It has been suggested that the therapeutical effect of flavonoids in the brain may be due to the ability of these flavonoids to interact with different neuronal and glial signalling pathways like Akt, PK-C and MAPK^38, 41, 42^. Moreover, flavonoids have been reported to inhibit the action of kinases involved in hyperphosphorylation of APP and tau and deter the abnormal processing of these proteins^43^. Similarly, in case of cancer studies results shown that flavonoids regulate many signalling pathways involved in cancer like NF-κB, MAPK, Wnt and mTOR which regulate cell survival and proliferation^25, 44, 45^. In addition to its neuroprotective attributes, many polyphenols such as flavonoids also possess antioxidative and antiproliferative activity; therefore providing cytoprotection against oxidative stress and induces apoptosis in cancer cells^46^.

It is known that polyphenols and flavonoids decreases cell viability and induces apoptosis in many prostate and breast cancer cell lines^41, 47–49^. These plant-based phenolic compounds target AMPK, PK-A, Akt and MAPK pathways in different organs like pancreas, muscle, liver and white adipocytes where they affect the glucose homeostasis and control diabetes^50, 51^. But, these therapeutic effects can’t be generalized as some studies have also reported inconclusive and even harmful results^52–54^. Therefore, it remains a subject of study since benefits of flavonoids are restricted to its subclasses and population subgroup under study^27, 55^. Polyphenols and flavonoids possess the above-mentioned potential therapeutic effects and which are well known in this class of phenolic compounds are selected and evaluated as inhibitors of MARK4.

In this study, apart from studying the binding of quercetin and its glucoside rutin with MARK4, we also selected ferulic acid, hesperidin, vanillin and gallic acid because these are most commonly used natural compounds in different applications^20, 56–59^. Molecular docking was performed to ascertain the interaction between these dietary polyphenols and MARK4. Docking results show that these compounds bind with MARK4 significantly. Fluorescence quenching and isothermal titration calorimetry (ITC) showed rutin and vanillin binds with MARK4 efficiently. Moreover, cytotoxicity and antiproliferative properties of rutin and vanillin were checked by MTT assay. Annexin-V, DHE staining and reactive oxygen species (ROS) determination was carried out to see the apoptotic and antioxidant potential of rutin and vanillin. *In vitro* kinase activity of MARK4 has been evaluated by cell free ATPase inhibition assay and in cell culture system by tau-phosphorylation assay. The results obtained confirm that all studied compounds interact with MARK4 but rutin and vanillin, shows very efficient binding with MARK4 and significantly inhibits its activity. Thus, these natural compounds can be considered as a potential inhibitor for MARK4.

## Results

### Selected natural compounds shows binding with MARK4

Polyphenols are known for their anticancer, antidiabetic and antiproliferative ability were used for docking with MARK4 to see possible interactions. Docking analysis helps to estimate the interacting residues, binding energies and intermolecular distance between the interacting MARK4 residues to the ligands. On the basis of interacting residues and binding energy obtained from docking analysis, the best-docked complexes were rutin, a quercetin derived glucoside and vanillin (Table 1).

**Table 1:** Binding parameters of selected natural compounds with MARK4 obtained from fluorescence and docking studies

S.No.	Flavonoids	Structure	∆*G* ^*^(kcal/mol)	^¶^Binding affinity (*K*_a_), M^−1^	Number of binding sites (*n*)
1	Gallic acid		−5.6	7.6	1
2	Quercetin		−8.6	1.02 × 10^2^	1
3	Hesperidin		−8.6	1.12 × 10^2^	1
4	Ferulic acid		−6.1	1.30 × 10^4^	1
5	Vanillin		−7.3	1.6 × 10^5^	1
6	Rutin		−9.3	2.87 × 10^5^	1

^*^Free energy calculated from docking analysis.

^**¶**^Binding affinity calculated from fluorescence quenching analysis.

**Figure 1:**
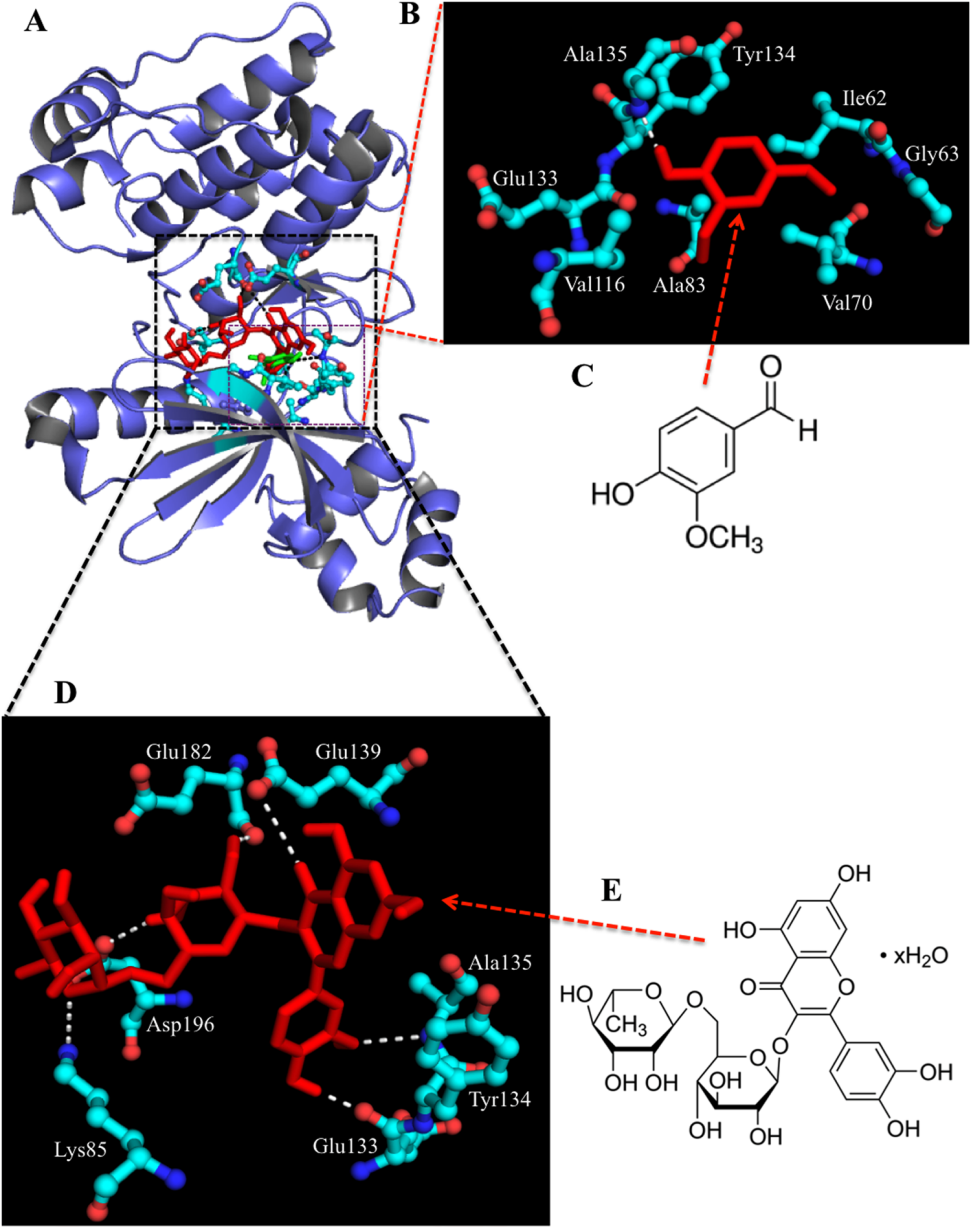
Molecular docking of rutin and vanillin with MARK4. **(A)** Binding of rutin (red) and vanillin (green) with MARK4 is shown in the cartoon model. **(B**) Interacting residues of MARK4 (ball and stick model) and vanillin (stick model). **(C)** Chemical structure of vanillin. **(D)** Interacting residues of MARK4 (ball and stick model) and rutin (stick model) **(E**) Chemical structure of rutin.

**Figure 2:**
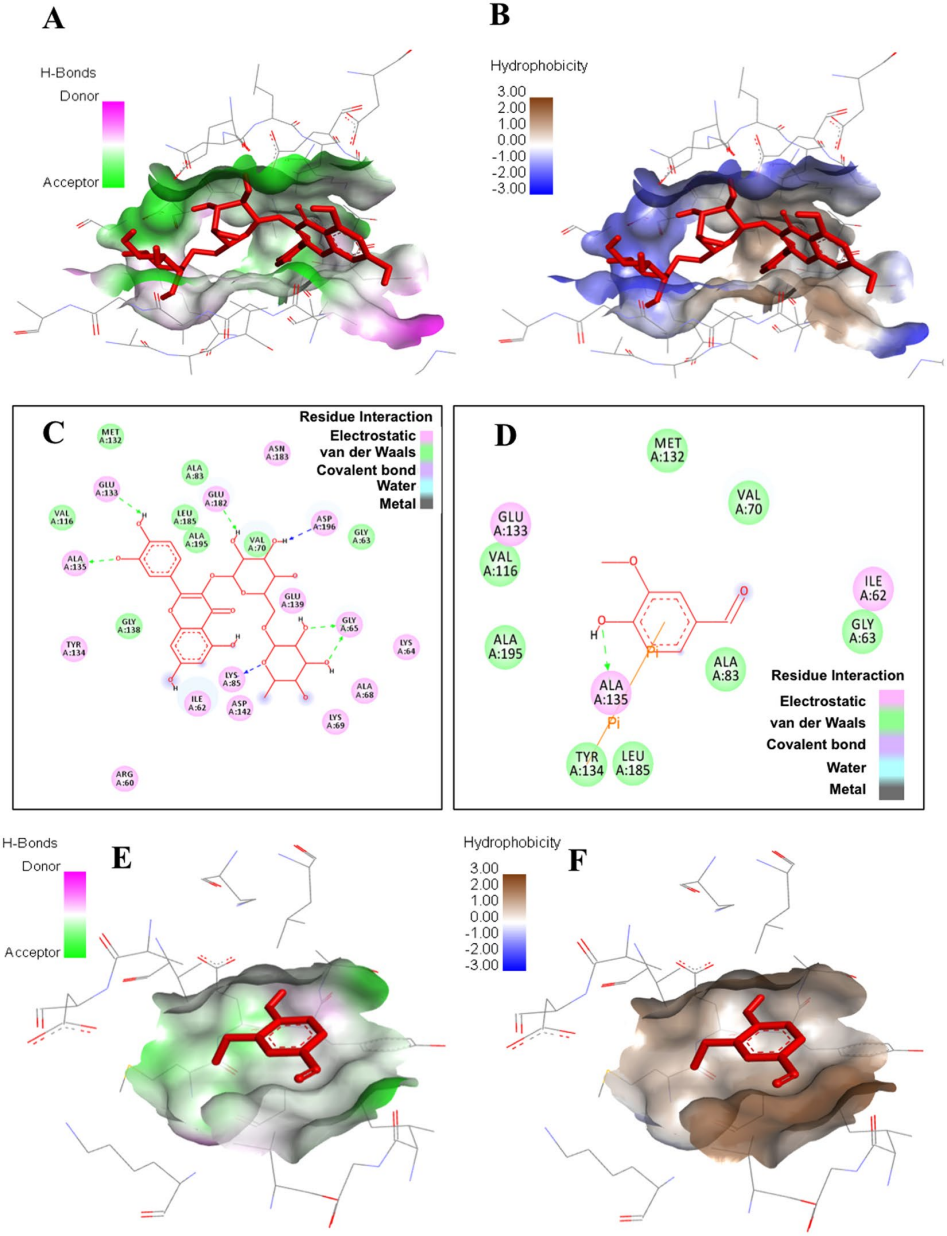
Molecular docking studies of rutin and vanillin: **(A,B)** Pocket view of MARK4 binding with rutin shows the hydrogen bond donor-acceptor residues and hydrophobic surface, respectively. **(C)** 2D schematic diagram of docking model of rutin with MARK4. Residues involved in hydrogen bonding, charge or polar interactions, van der Waals interactions are represented by respective colour indicated in inset of figure. (**D**) 2D schematic diagram of docking model of vanillin with MARK4. Residues involved in hydrogen bonding, charge or polar interactions, van der Waals interactions are represented by respective colour indicated in inset of figure. **(E,F**) Pocket view of MARK4 binding with vanillin shows the hydrogen bond donor-acceptor residues and hydrophobic surface, respectively.

Major residues of MARK4 that interact with most of the studied ligands are Gly65, Lys85, Glu133, Tyr134, Ala135, and Asp196. Rutin interacts to MARK4 by forming hydrogen bonds with Lys85, Glu133, Ala135, Glu182 and Asp196 at distances 3.2 Å, 2.0 Å, 3.0 Å, 2.2 Å and 2.4 Å, respectively (see Fig. 1 and supplementary Fig. S1). Besides such strong hydrogen bonding, rutin forms several other interactions to Ile62, Gly63, Lys64, Ala68, Lys69, Val70, Ala83, Val116, Met132, Gly138, Asp142, Asn183, Leu185 and Ala195 **(**Fig. 2 **)**. Although vanillin offers only single hydrogen bond (3.3 Å) to MARK4, exists between Ala135 and hydroxyl group of vanillin, but along this it also forms a π-π bond with Tyr134 **(**Fig. 2D **)**. Other residues of MARK4 named Ile62, Gly63, Val70, Ala83, Val116, Met132, Leu185 and Ala195 interacts with vanillin (see Fig. 2D and supplementary Fig. S2). The interaction of ferulic acid involved formation of three hydrogen bonds with Lys85 and Ala135 and Asp196. Three hydrogen bonds were formed between quercetin and MARK4 including residues Lys85, A135 and Asp196. The hydrogen bond forming residues in case of hesperidin were Lys64 and Glu103. Gallic acid also showed weak binding and formed hydrogen bonds with Ile62 and Ala135 (3.0 Å) (see supplementary Fig. S3).

### Fluorescence binding studies

**Figure 3:**
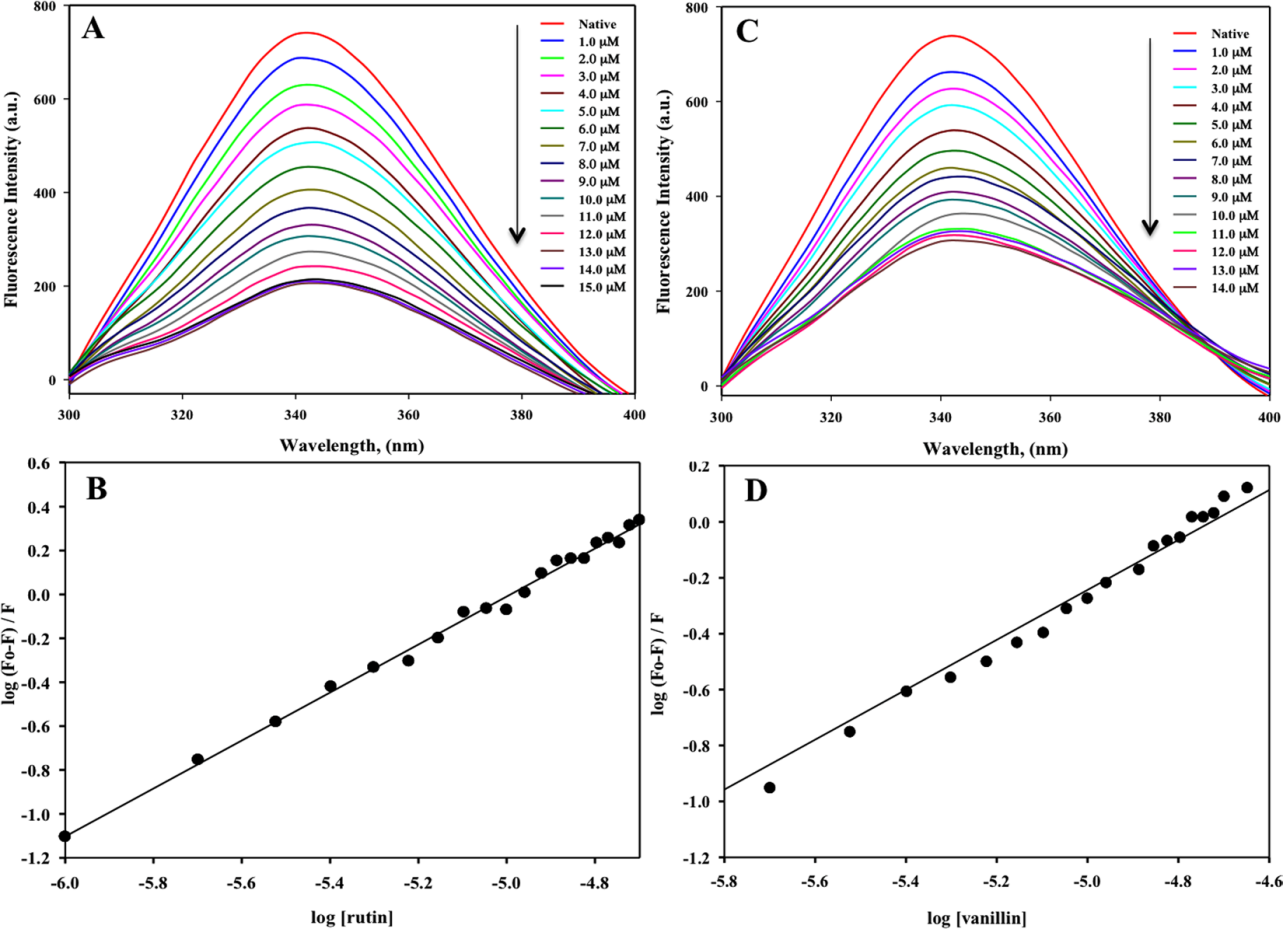
Binding studies of rutin and vanillin with MARK4 using fluorescence spectroscopy. **(A)** Fluorescence spectra of MARK4 (5 µM) with increasing concentration of rutin (0–15 µM). Excitation wavelength was fixed to 280 nm and emission was recorded in the range 300–400 nm. **(B)** Modified Stern-Volmer plot showing quenching of MARK4 by rutin used to calculate binding affinity (*K*_a_) and number of binding sites (*n*). **(C)** Fluorescence spectra of MARK4 (5 µM) with increasing concentration of vanillin (0–14 µM). Excitation wavelength was fixed to 280 nm and emission was recorded in the range 300–400 nm. **(D)** Modified Stern-Volmer plot showing quenching of MARK4 by vanillin.

In order to validate docking results, we used fluorescence spectroscopy to measure the binding affinity of ligands to MARK4 using Trp as probe. MARK4 was successfully cloned, expressed and purified. For each measurement, 5–10 µM protein was taken in a quartz cuvette and titrated by 1.0 µM of selected natural polyphenols from a 1.0 mM stock. The protein was excited at λ_280_ nm and emission was recorded within the range of 300–400 nm. We have screen all docked compounds by fluorescence quenching measurements. To obtain the saturation point we have titrated the MARK4 with increasing concentrations of respective compound, the concentration was varied from 0–100 µM. Compounds possesses good binding affinity shows the saturation at low concentrations whereas the compounds which does not show binding don’t quench even at high concentration (100 µM). For clarity and easy interpretations of results we have shown the refine spectrum of fluorescence binding studies. The best quenching on addition of increasing amount of ligand was observed in case of rutin and vanillin **(**Fig. 3A,C). For others also, quenching was observed but not so appreciable (see supplementary Fig. S4). The fluorescence result was in accordance with the docking results.

The inverse relationship between fluorescence intensity and the concentration of each natural polyphenols has been described by Stern-Volmer equation, in which *K*_a_ and *n* represents binding constant and number of binding sites per protein molecule, respectively. We observed that rutin is having the highest binding affinity of 2.87 × 10^5^ M^−1^ and possess single binding site (Fig. 3B). Vanillin also showed a good binding with *K*_a_ calculated as 1.65 × 10^4^ M^−1^ (Fig. 3D). However, other studied natural polyphenols did not show any appreciable binding. Thus, the above finding suggests that rutin and vanillin are best among all other natural compounds used in this study and they can be used as good inhibitor against MARK4 **(**Table 1 **)**.

### Enzyme inhibition assay

**Figure 4:**
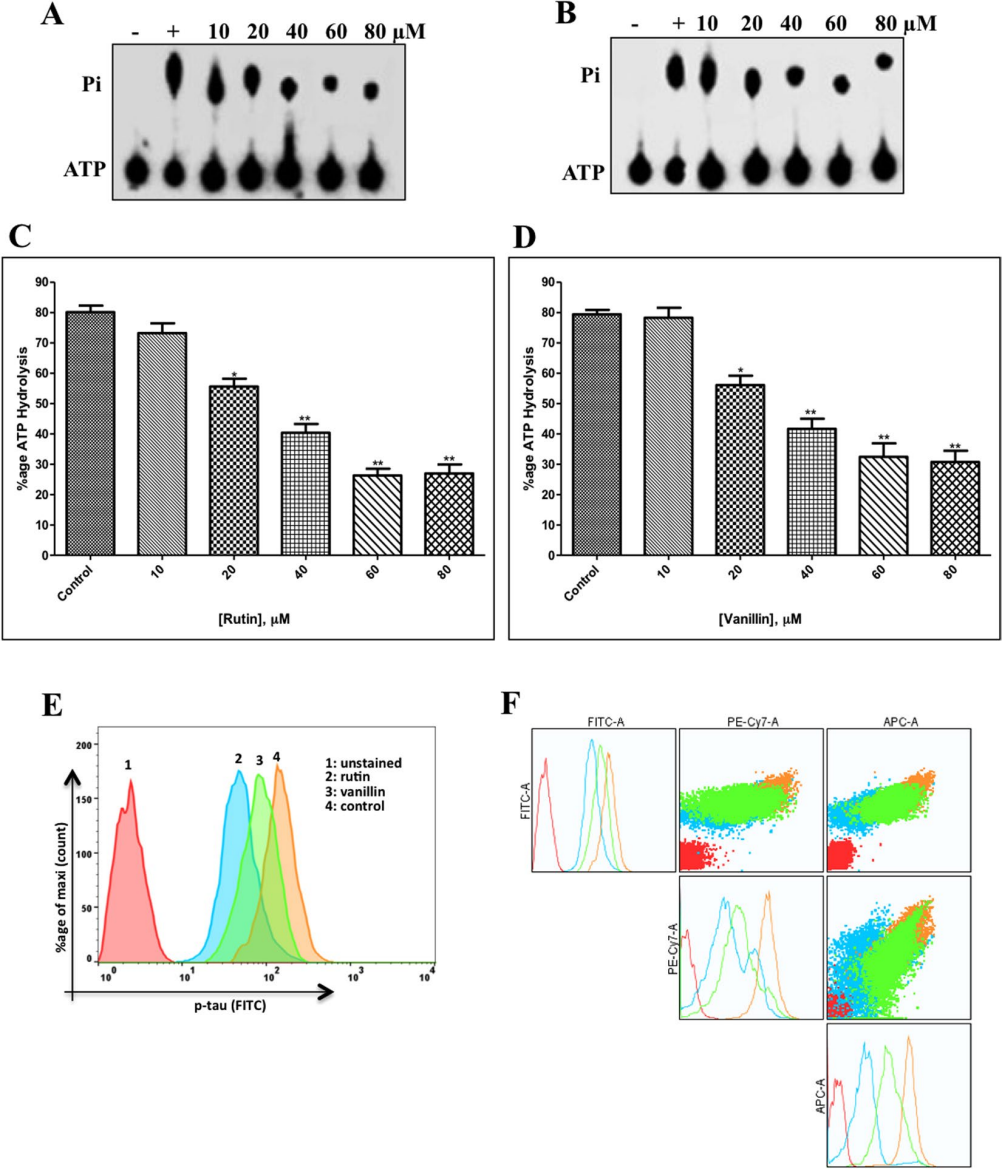
ATPase enzyme inhibition and tau-phosphorylation assay of MARK4. **(A,B)** Shows the hydrolysis of Pi from ATP, position of Pi and ATP spots are indicated on left side. Lane 1, negative control (without protein); lane 2, 100 nM MARK4 (positive control); and lanes labeled as 10,20,40,60 and 80, shows the concentration of rutin and vanillin in µM incubated with 100 ng MARK4, respectively. **(C,D**) ATPase inhibition (% hydrolysis of ATP) with increasing concentrations of rutin and vanillin are shown in bar diagram. Bar graph represents the normalized intensity of ATP hydrolysis for duplicate measurements ± SD. *p < 0.05; **p < 0.01, compared with the control. Statistical analysis was done using Student’s t- test for unpaired samples. (**E**) Representative flow cytometry histogram of SH-SY5Y cell fractions stained with phosphorylated anti-tau antibodies, each histogram represents the phosphorylation status of tau under different treatments as mentioned in inset (**F**) NXN plot of SH-SY5Y cells treated with rutin and vanillin stained with phosphorylated anti-tau. It represents the multiple parametric analysis of cell fractions under different treatments stained with phosphorylated anti-tau.

ATPase inhibition assay was performed for screening of all the selected natural compounds as an inhibitor of MARK4. We checked the ATPase activity of MARK4 in the presence of all natural compounds. During initial screening, a significant decrease in the enzyme activity was seen in the presence of rutin and vanillin (see supplementary Fig. S5). Further enzyme inhibition assay of MARK4 was performed with increasing concentrations of rutin and vanillin (10–80 μM) as shown in Fig. 4A,B. These enzyme inhibition results show that at 40 μM concentrations both rutin and vanillin inhibited ATPase activity nearly by 50% (Fig. 4C,D). These results suggest that among studied compounds rutin and vanillin acts as potential inhibitor of MARK4.

### Tau-phosphorylation assay

Cell free *in vitro* ATPase inhibition assay suggested that rutin and vanillin inhibit the MARK4 activity significantly. In order to confirm this observation, enzyme inhibition activity is extended to a cell system based tau-phosphorylation assay. Tau proteins act as the substrate for MARK4 mediated phosphorylation. To see the functional consequences of MARK4 inhibition by rutin and vanillin, SH-SY5Y cells were allowed to grow in presence of rutin and vanillin and subsequently phosphorylation of tau has been assessed with the help of flow cytometry. It was found that rutin and vanillin inhibit the phosphorylation of tau (as shown in Fig. 4E,F). Result presented in the Fig. 4E shows that treatment of rutin and vanillin shifts the position of histogram towards the lower side of untreated cells (shown by 4: control). It is of worth mentioning that as compared to vanillin, rutin inhibits the phosphorylation of tau to a greater extent (as shown by histogram 2 in Fig. 4E). Panel F of the Fig. 4 represents the NXN plot of cells stained with anti-p-tau. It was clear from this plot that when phosphorylation pattern of tau was compared according to multiple parameters as labeled on each axis, it also follows the same pattern of phosphorylation. Here, NXN plot helps to visualize the corresponding change in fluorescence in N dimensions, Fig. 4F gives the respective change in tau-fluorescence (labeled with anti-p-tau) of SH-SY5Y cells treated with rutin and vanillin. This data supports our histographic multiple overlay shown in Fig. 4E, that when we compare the phosphorylation status of tau in presence of rutin and vanillin in respective dimensions, it shows a considerable decrease in phosphorylation of tau. It can be easily observed from Fig. 4F (in both histogram and dot plots) the phosphorylation status of tau in vanillin treated cells (shown by sky blue colour), rutin treated cells (shown by parrot green colour) is found to be decreased as compared to the untreated control cells (shown by pale yellow colour). These results are clearly suggesting that rutin and vanillin inhibits the phosphorylation of tau.

### ITC measurements

**Figure 5:**
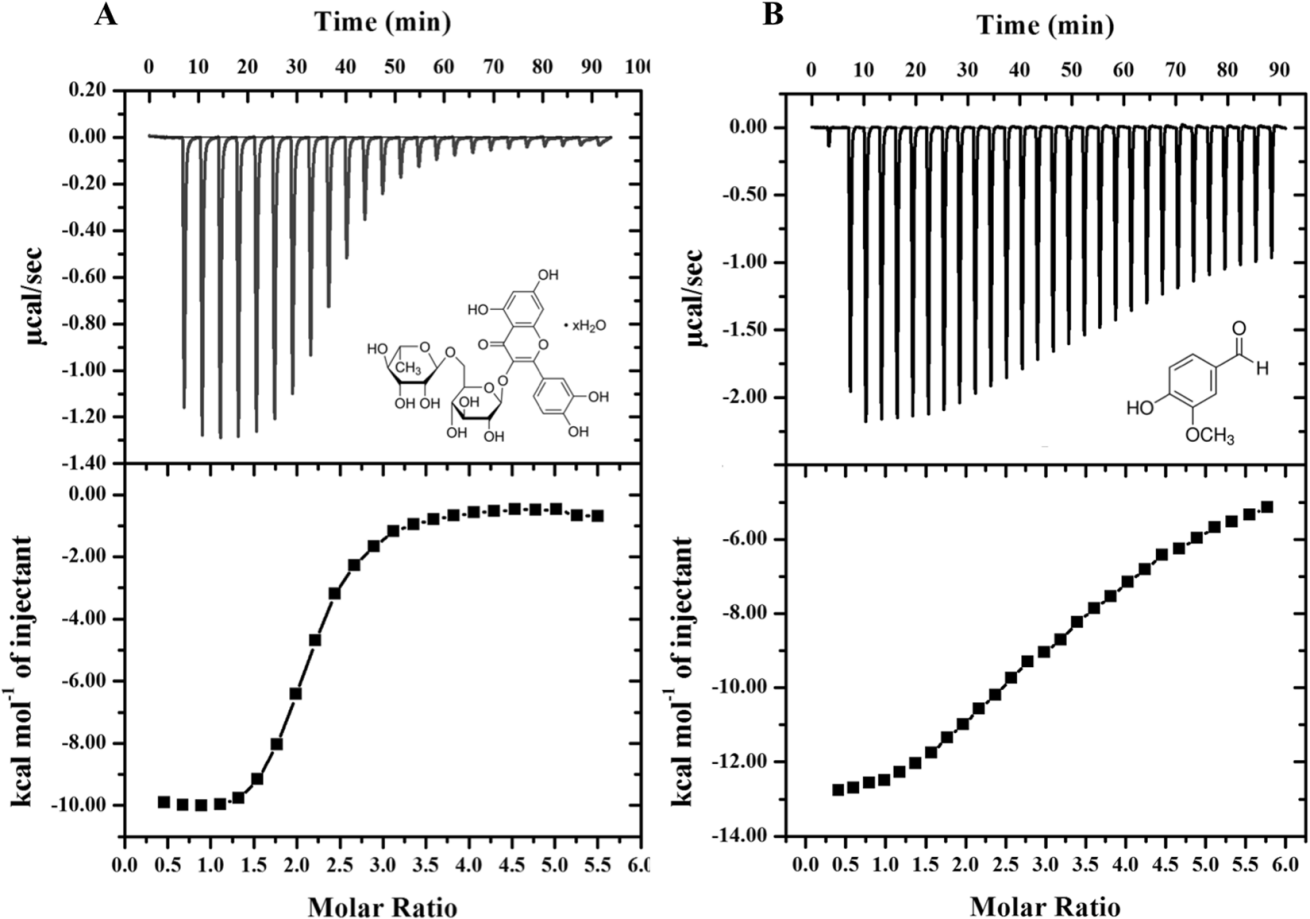
ITC measurement showing the titration of rutin and vanillin with MARK4. (Top) Raw data plot of heat produced against time for the titration of 800–1200 µM rutin and vanillin into 14–20 µM MARK4. (Bottom) Corresponding binding isotherm obtained after integration of peak area and normalization to yield a plot of molar enthalpy change against rutin/vanillin-MARK4 ratio. The two-site fit curve is displayed as a thin line. Experiments were done in duplicate.

We observed that the rutin and vanillin showing strong binding and reduces enzyme activity of MARK4. To ascertain high binding affinity and associated thermodynamic parameters, we did ITC measurements of rutin and vanillin binding to the MARK4. A typical ITC isotherm obtained from titration of rutin and vanillin with MARK4 is shown in the Fig. 5. The upper section shows raw data with negative heat pulses indicating exothermic binding. After subtracting the dilution heats of both compounds and protein gives the integration area and binding curves which are shown in the bottom panels of Fig. 5. This gives the extent of heat produced corresponding to the each injection as a quantity of the molar ratio of studied compound to that of MARK4. The results presented were obtained from two-site model of fitting. Different thermodynamic parameters associated with binding of rutin and vanillin with MARK4 (Δ*H*, enthalpy change and Δ*S*, entropy change) is shown in Table 2. ITC Results of both rutin and vanillin shows a very good binding with MARK4.

**Table 2:** Thermodynamic parameters obtained from the calorimetric titration of rutin and vanillin with MARK4.

Compound	*K*_a_ (association constant), M^−1^	*K*_D_ (dissociation constant) μM	Δ*H* (enthalpy change), cal/mol	Δ*S*, cal/mol/deg
Rutin	*K*_a1_ = 2.04 × 10^5^ ± 0.15 × 10^5^	*K*_D1_ = 4.9	Δ*H*_1_ = −4.61 × 10^6^	Δ*S*_1_ = −1.52 × 10^4^
	*K*_a2_ = 1.72 × 10^6^ ± 0.31 × 10^6^	*K*_D2_ = 0.581	Δ*H*_2_ = −1.0 × 10^4^	Δ*S*_2_ = −4.74
Vanillin	*K*_a1_ = 8.43 × 10^3^ ± 0.5 × 10^3^	*K*_D1_ = 118.6	Δ*H*_1_ = −4.90 × 10^5^	Δ*S*_1_ = −1.60 × 10^3^
	*K*_a2_ = 1.45 × 10^5^ ± 0.29 × 10^5^	*K*_D2_ = 6.9	Δ*H*_2_ = −1.22 × 10^4^	Δ*S*_2_ = −16.9 × 10^4^

### Cell proliferation assay

**Figure 6:**
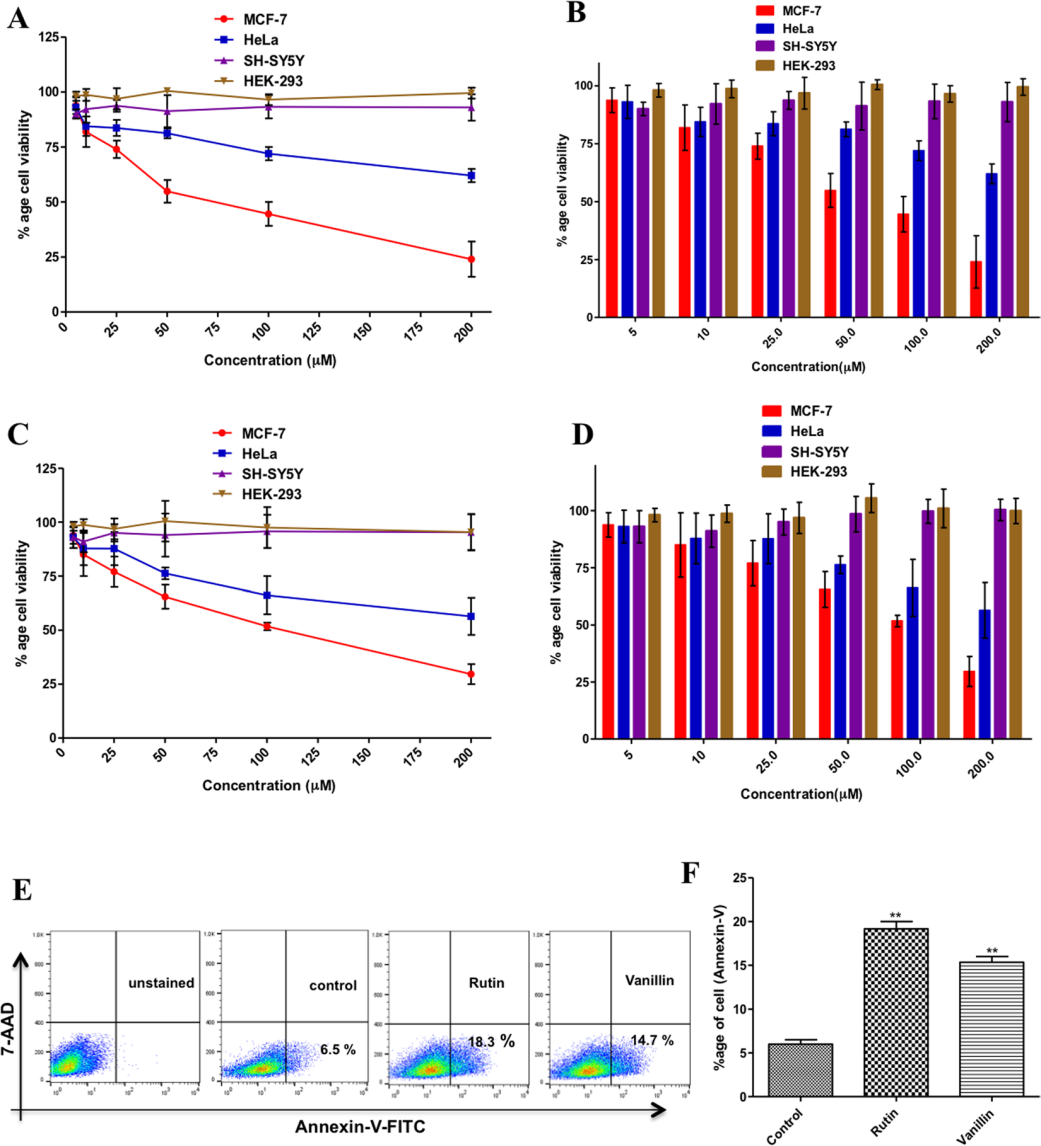
Cell proliferation, viability and apoptosis studies. **(A–D**) Effect of rutin and vanillin on the viability of different cancer and normal cell lines: Cells were treated with increasing concentrations of rutin and vanillin (0–200 μM) for 48 h. Cell viabilities were shown as a percentage of the number of viable cells to that of the control. Values > 100 shows the percentage increase in cell survival to that of control (untreated cells). Each data point shown is the mean ± SD from n = 3. **(E**) Annexin-V staining of MCF-7 cells; cells were treated with IC_50_ concentrations of rutin (80 μM) and vanillin (120 μM) for 48 h and subsequently stained with FITC-Annexin-V. Stimulation of apoptosis was quantified by flow cytometry. Representative flow images showing FITC-Annexin-V labeled cells, which directly corresponds to the percentage of apoptotic cells. **(F**) Bar graphs represents the percentage of apoptotic MCF-7 cells stained with Annexin-V for duplicate measurements ± SD. **p < 0.001, compared with the control (untreated cells). Statistical analysis was done using one-way ANOVA and t-test for unpaired samples. For anticancer activities doxorubicin has been taken as positive control.

We performed 3-[4,5-dimethylthiazol-2-yl]-2,5-diphenyl tetrazoliumbromide (MTT) assay to see the effects of rutin and vanillin on cell viability of human breast cancer (MCF-7), human cervical cancer (HeLa), neuronal (SH-SY5Y) and nontumorigenic human embryonic kidney (HEK293) cells. MCF-7, SH-SY5Y and HEK-293 cells are taken as these cells/tissues from which these cells has been derived are having reported expression of MARK4 and in MCF-7 cells role of MARK4 has been evaluated for various biological functions^4, 7, 10, 11^. Both rutin and vanillin treatment inhibited cell viability of MCF-7 and HeLa cells in a dose-dependent manner **(**Fig. 6A–D **)**. The 50% inhibitory concentration (IC_50_) of rutin and vanillin for MCF-7 cells is 80 μM and 120 μM, respectively, whereas for HeLa cells it is >200 μM. For doxorubicin (taken as positive control for cell proliferation studies), the IC_50_ for MCF-7 cells is 0.12 μM. Interestingly, treatment of rutin and vanillin both, at a dose even higher than as 200 μM, did not inhibit the viability of HEK293 and SH-SY5Y cells (Fig. 6A–D). Contrary to MCF-7 and HeLa cells, in case of HEK293 and SH-SY5Y cells treatment of rutin and vanillin supports the cells viability (shown in Fig. 6A–D).

### Apoptosis assay

Apoptosis plays a central role in the progress and pathophysiology of a wide variety of diseases. However, due to impairment of apoptotic signal transmission most of the cancerous cells evade apoptosis. A probability was investigated whether decrease in MCF-7 cells viability by the treatment of rutin and vanillin is due to the induction of apoptosis. Annexin- V staining was used to study the apoptosis. For this experiment (shown in Fig. 6E), cells were serum-starved and incubated with IC_50_ concentrations of rutin and vanillin for 48 h. Cells were washed twice with phosphate-buffered saline (PBS) and stained with FITC labeled Annexin- V. Annexin- Vpositive cells were examined by flow cytometry. It was observed that treatment of rutin (IC_50_ = 80 μM) and vanillin (IC_50_ = 120 μM) induces apoptosis in MCF-7 cells (Fig. 6E). Quantification by flow cytometry analysis shows that treatment of rutin (80 μM) stimulated a 12% increase in apoptosis of MCF-7 cells and vanillin increases apoptosis by 9% as compared to the control cells (Fig. 6E).

### Estimation of reactive oxygen species levels

**Figure 7:**
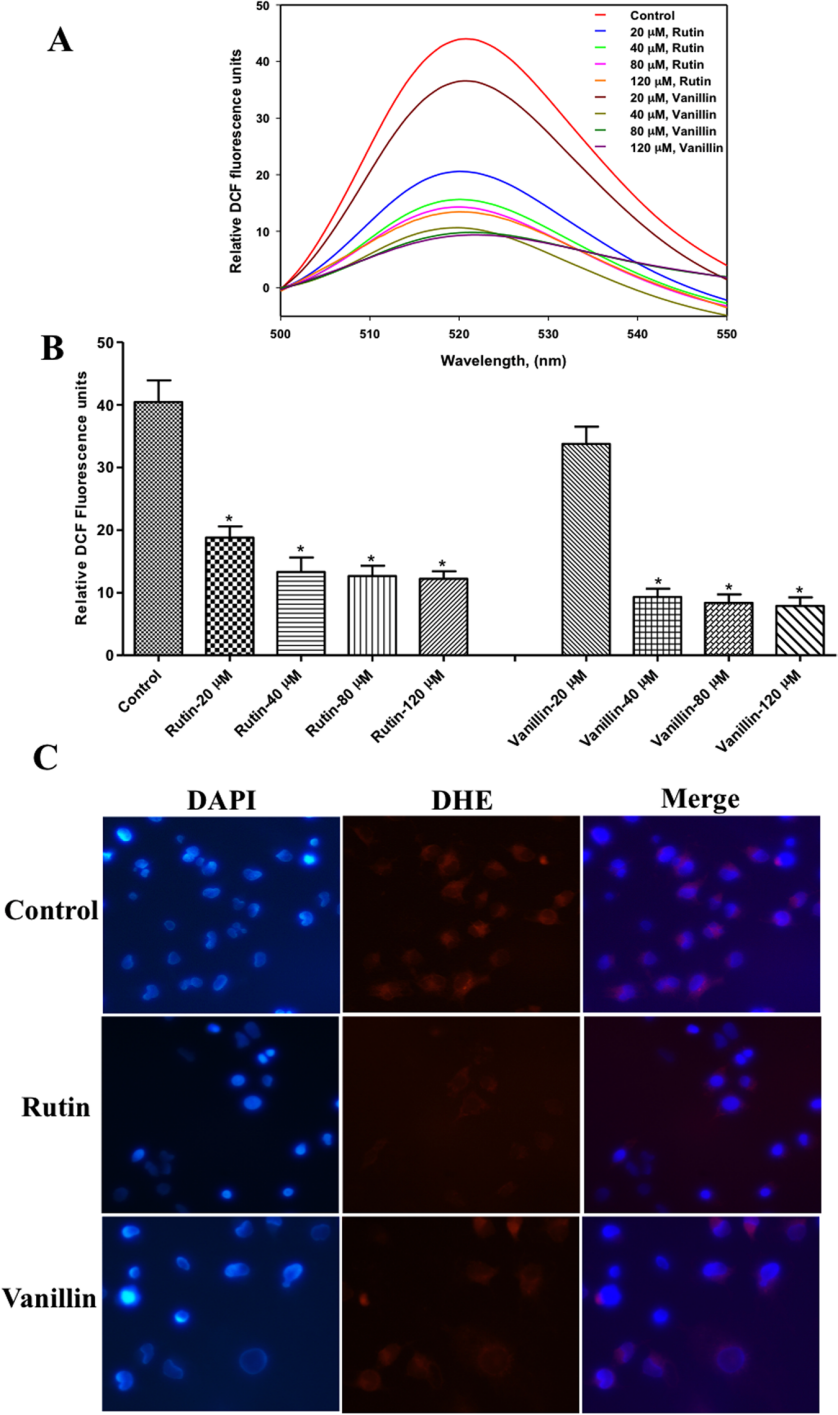
Effect of rutin and vanillin on the production of ROS. **(A)** Fluorescence emission spectrum of DCF probe, MCF-7 cells was treated with increasing concentrations of rutin and vanillin. Stained with DCFDA dye and DCF fluorescence was measured by spectroflourimeter. **(B)** Bar graph presentation of DCF fluorescence with respective treatment. Bar graph represents the relative intensity of DCF fluorescence for duplicate measurements ± SD. *p < 0.05, compared with the control (untreated cells). Statistical analysis was done using one-way ANOVA and t-test for unpaired samples. **(C**) Representative images of MCF-7 cells stained with DHE for the assessment of cytoplasmic superoxide levels after the treatment of rutin (IC_50_ = 80 μM) and vanillin (IC_50_ = 120 μM). After the incubation of cells with rutin and vanillin they were subsequently stained with DAPI and DHE as labeled. Red fluorescence shows the presence of superoxides and intensity of red colour represents the levels of superoxides.

In this assay, MCF-7 cells were treated with increasing concentrations of rutin and vanillin (20–120 μM) for 12 h (Fig. 7). Followed by incubation, to measure intracellular ROS, 2-Dichlorofluorescein diacetate (DCFDA) staining was performed. Treatment of rutin and vanillin resulted in a dose-dependent decrease in DCF fluorescence. However, treatment of rutin and vanillin at a concentration of >80 μM induces a slight change in DCF-fluorescence (Fig. 7A,B **)**. These results indicate a decrease in intracellular ROS levels in rutin and vanillin treated MCF-7 cells. Further, cytoplasmic superoxide level was measured by dihydroethidium staining (Fig. 7C). After treating with rutin (80 μM) and vanillin (120 μM), MCF-7 cells were stained with DHE and examined by fluorescence microscopy. In fluorescence imaging, substantial decreases in the extent of DHE fluorescence as compared to control (untreated cells) have been observed (Fig. 7C **)**. It shows that treatment of rutin and vanillin decreases cytoplasmic superoxide levels. These results imply the antioxidant behavior of rutin and vanillin.

## Discussion

One major problem in drug designing and chemical synthesis is the cytotoxicity of synthesized compound to that of normal cells. Plant-based natural products have therapeutic potential and in most of the cases non-cytotoxic in nature; therefore natural products or phytonutrients are the better options to explore in the field of drug designing. Natural dietary polyphenolic compounds have been established as significant anticancer and neuroprotective therapeutic agents due to their potential of tumor growth inhibition, angiogenesis, metastasis, induction of apoptosis in cancer cells and neuroprotection without parting major side effects^44–46^. These important therapeutic behaviors are generally shown by flavonoids and they have been reported to inhibit the action of kinases involved in hyperphosphorylation and abnormal processing of different proteins like APP and tau^43, 60^. MARK4 is a potential drug target as it linked with Alzheimer’s disease because it phosphorylates tau protein^9–11, 61^. Besides neurological disorders, several studies established the role of MARK4 in breast, liver, prostate cancer progression, occurrence of type-II diabetes and several metabolic ailments^4, 5, 7, 8, 62^. Due to the immense importance of dietary flavonoids and other natural antioxidants, we evaluate them as potential inhibitors for MARK4. After the initial screening by docking and fluorescence studies, role of two natural compounds rutin and vanillin as an inhibitor of MARK4 has been studied in detail. Rutin is a dietary flavonoid and vanillin is a phenolic aldehyde, both are present in our routine foodstuffs like citrus fruits and vegetables^22, 57, 59^. Here we investigate their role in MARK4 inhibition.

The docking analysis revealed that rutin and vanillin showed major interaction and strong binding with MARK4 as compared to other natural polyphenols. The rutin-MARK4 complex is mainly stabilized by hydrogen bonding interaction (six H-bonds) whereas vanillin-MARK4 complex is stabilized by hydrogen bonding as well as π-π interactions **(**Figs 1 and 2 **)**. From these docking studies we have drawn an important observation that all of the studied polyphenols binds to the previously known binding cavity of MARK4 to which its substrate binds. It means that the studied compound decreases the affinity of MARK4 for its substrate and behaves as inhibitors. Fluorescence binding studies shown in Fig. 3 suggests that although both rutin and vanillin binds efficiently with MARK4, but in comparison to vanillin, binding of rutin is stronger.

ATPase and tau-phosphorylation assays further validates these results, which depicts that in comparison to other studied compounds both rutin and vanillin significantly inhibits the activity of MARK4 **(**Fig. 4). One important inference from the tau-phosphorylation inhibition helps to explain the better survival of studied neuronal and kidney cells. As in case of MARK overexpressing neuronal cells, tau is found to be hyperphosphorylated, which leads to the abnormal processing of tau^9, 11^. Our results supports this notion that inhibition of MARK4 down regulate the phosphorylation of tau and supports the growth of SH-SY5Y neuronal cells. So here inhibition of MARK4 may be possible reason that supports growth of neuronal cells. Finally, thermodynamic parameters of binding were determined by ITC, the values of association/dissociation constant are shown in Table 2, depicts consistent behavior of rutin and vanillin with MARK4. It also suggests that both rutin and vanillin binds with MARK4 efficiently but binding of rutin is stronger as compared to vanillin. We have further attempted to work out on the potential of rutin and vanillin as an anticancerous molecule by investigating its antiproliferative and apoptotic action on MCF-7 cells. Cellular toxicity and viability analysis indicates that rutin and vanillin inhibits the growth of MCF-7 cells significantly in a dose dependent manner (Fig. 6). They also inhibit the proliferation of HeLa cells, but to a lesser extent. But in case of HEK293 and SH-5S5Y cells these natural compounds supports their survival, as normally in these cell lines tau is found to be hyperphosphorylated and these molecules inhibits the MARK4 activity that lower down the phosphorylation of tau, which might be the reason for their better growth. Annexin-V is early stage apoptotic marker, staining by Annexin-V suggest that treatment of rutin and vanillin induces apoptosis in MCF-7 cells **(**Fig. 6). MARK4 is previously known to inhibit hippo signalling in breast cancer cell^4^ and acts as the negative regulator of mTORC1^15^ also, both of these pathways are responsible for proliferation and migration of cancer cells. So any molecule or inhibitor that inhibits MARK4 can be responsible to regulate these pathways. Here our results are in consistency with earlier reports that inhibition of MARK4 reduces that cell proliferation and induces apoptosis in MCF-7 cells. Further reduction in ROS and cytoplasmic superoxide level supports antioxidant behavior of rutin and vanillin^63, 64^. MARK4 is known to induces oxidative stress in adipocytes^5^, so inhibition of MARK4 may lead to the reduction of ROS and helps to relive oxidative stress. Rutin and vanillin both inhibits the activity of MARK4 and also reduces the levels of cellular ROS. Our observations of MARK4 inhibition by studied compounds and reduction in ROS also in accordance with the results of Liu *et al*.^5^, which suggested that inhibition of MARK4 reduces oxidative stress. This antioxidant property of these compounds is a good sign for normal cells and bad for cancerous cells. Inhibition of cell proliferation, antioxidant behavior and induction of apoptosis in MCF-7 cells clearly indicates the anticancerous behavior of rutin and vanillin. Other important observation from the cell proliferation assays on HEK293 and neuronal cells is the improvement in the survival of these cells by the treatment of rutin and vanillin. This observation further supports the application of dietary flavonoids or antioxidants for combating the neurological disorders.

In conclusion, this study confirms the binding of rutin and vanillin with MARK4 and suggesting these natural compounds as potential inhibitors for MARK4. Furthermore, our observations imply that targeting of MARK4 by rutin and vanillin may be an efficient approach to combat with the pathophysiology of cancer and neurodegenerative disorders. Results from this study advocate the use of natural/dietary compound in the area of inhibitors/drug development against MARK4 or other kinases.

## Materials and methods

### Materials

CAPS, N-lauroyl sarcosine, MTT (3-[4,5-dimethylthiazol-2-yl]-2,5-diphenyl tetrazolium bromide), rutin hydrate, hesperidin, vanillin, quercetin, ferulic acid, gallic acid (approx. 95%) and other reagents were purchased from Sigma Aldrich (St. Louis, MO). Ni-NTA column and gel filtration column (Superdex-75) were purchased from GE healthcare (GE Healthcare Life Sciences, Uppsala, Sweden). HeLa (human cervical cancer cells), MCF-7 (human breast cancer cells), SH-SY5Y (human neuroblastoma cells) and HEK293 (human embryonic kidney cells) cells was procured from National Centre for Cell Sciences (NCCS), Pune, India. Anti-tau(pSer262) cat:44–750 G, FITC labeled goat anti-rabbit IgG (31635) secondary antibodies and dihydroethidium were taken from Invitrogen, Thermo Fisher Scientific. FITC-Annexin-V detection kit was purchased from BD-Pharmingen, BD Biosciences (USA). Dulbecco minimal essential medium (DMEM), RPMI-1640 and Ham’s F-12 nutrients mix cell culture medium and fetal bovine serum (FBS) were purchased from Gibco life sciences. All reagents used were of molecular biology grade.

### Expression and purification of MARK4

MARK4 (amino acid residues 59 to 368) was successfully expressed in M15 competent cells and was then purified using our reported method with some modifications^16, 65, 66^. In brief, the recombinant cells were grown and induction was done at 16 °C by minimum concentration of IPTG i.e. 1 mM. The pellet obtained from this culture was dissolved in lysis buffer (50 mM Tris, 20 mM EDTA, 0.1 mM PMSF and 1% Triton-100) and inclusion bodies were prepared. Further, inclusion bodies were dissolved in sarcosine buffer (50 mM CAPS, 1.5% N-laurosyl sarcosine, pH 11.0) and were centrifuged for 25 min at 12,000 rpm and the supernatant was collected. The supernatant so obtained was allowed to bind on Ni-NTA column (Qiagen QIA express). Washing followed this step with 5 mM imidazole in sarcosine buffer. Elution was done with increasing concentration of imidazole from 10 mM to 400 mM. The purity of elutant was then checked on SDS-PAGE.

### Molecular docking

The atomic co-ordinate of MARK4 available in the protein data bank (www.rcsb.org, PDBID: 5ES1), was optimized using steepest descent method from Gromacs 4.5.5.The 2D and 3D structures of all the natural compounds were regained from PubChem (https://pubchem.ncbi.nlm.nih.gov/compound/5281318#section=2D-Structure/3D-Conformer). Further calculations, file preparations are done according to our previously published protocol^3^. After preparing the coordinate files of MARK4 and respective compound, it was subjected to docking using AutoDock 4 package^67^. The interaction between MARK4 and the listed natural compounds was analyzed using the Lamarckian genetic algorithm (LGA). The binding energy was calculated using van der Waals, electrostatic interactions and hydrogen bonding. Finally docked complexes of MARK4 were further optimized, validated and explored using “Receptor–Ligand Interactions” modules of Discover Studio 4.0^68^. PyMOL were used for the visualization of molecular interactions exists in the final dock^69^.

### Fluorescence measurements

The binding study of ligand with protein was done by monitoring changes in fluorescence intensity of protein. Jasco spectroflourimeter (FP-6200) was used to carry out fluorescence experiments using a 5mm cuvette of quartz. The temperature was maintained as 25 ± 0.1 °C by using an external thermostated water circulator. The ligands were dissolved in DMSO, and diluted to 1 µM/µl working concentration in the phosphate buffer. The protein was excited at 280 nm and the intrinsic fluorescence emission spectra were recorded at 300–400 nm. The characteristic emission peak was seen at 346 nm. As recombinant MARK4 (residues 59–368) consists of two tryptophan residues, they absorb at 280 nm and give their characteristic emission maxima nearly at 346, that why we choose fluorescence quenching (due to binding of ligand with protein) experiments as a criteria to determine the binding. The final spectra were obtained by subtracting with the corresponding blank. The experiments were performed in triplicates and the average data was analysed. The decreased fluorescence intensity with increase in the concentration of ligand forms the basic criteria for deducing the binding constant (*K*_a_) as well as number of binding sites present on protein (*n*) using the modified Stern-Volmer equation^70^:

1 $$\mathrm{log}({{\rm{F}}}_{{\rm{o}}}-{\rm{F}})/{\rm{F}}=\,\mathrm{log}\,{{\rm{K}}}_{{\rm{a}}}+{\rm{n}}\,\mathrm{log}[{\rm{L}}]$$

where, F_o_ = Fluorescence intensity of native protein, F = Fluorescence intensity of protein in the presence of ligand, *K*_a_ = Binding constant, *n* = number of binding sites, L = concentration of ligand. The values for binding constant (K_a_) and number of binding sites (*n*) were derived from the intercept and slope, respectively.

### ATPase and tau-phosphorylation inhibition assay

ATPase assay was used to check the enzyme activity of MARK4 protein in the presence of different compounds; for this we used previously published protocol from our group^6, 71^. Briefly, we measured ^32^Pi released from [γ-^32^P] ATP hydrolysis, which was catalyzed by MARK4. After incubating proteins with ice-cold ATP (1 mM) and [γ-^32^P] ATP (specific activity 222 TBq mmol^−1^) for 2 h at 37 °C thin layer chromatography was performed. We first preceded our experiment in the presence of increasing concentrations of all selected natural compounds with MARK4. Finally, after initial screening, MARK4 were incubated with increasing concentrations of rutin and vanillin. These results were taken to measure the MARK4 inhibition in terms of percentage hydrolysis of ATP, using imageJ software (https://imagej.nih.gov/ij/index.html). For tau-phosphorylation inhibition assay, SH-SY5Y cells were grown in 6-well cell culture plate and treated with rutin and vanillin (80–120 μM). After 24 hrs, 1 × 10^6^ cells were harvested and staining was done within 1 h of harvest. Followed by three times washing with PBS containing 2%BSA cells were fixed with fixation buffer (2% paraformaldehyde) for 40 min (4 °C) and permeabilized with permeablization buffer (eBioscience) and 0.5% saponin for 30 min at 4 °C. The cells were washed two times, resuspended in incubation buffer and incubated with primary antibodies of anti-tau (pSer262), at 25 °C for 2 hr. After incubation cells were washes as before and labeled with FITC labeled goat anti-rabbit IgG secondary antibody for 30 min at room temperature. Flow cytometry analysis was performed on FlowJo (BD Bioscience, USA).) At least 10,000 events were acquired for each sample.

### Isothermal titration calorimetery

Isothermal titration calorimetery (ITC) measurements were done at 25 °C on a VP-ITC microcalorimeter from MicroCal, Inc (GE, MicroCal, USA). Protein was extensively dialyzed against 50 mM phosphate buffer and the ligand was dissolved in last dialyzing buffer. Equal amount of DMSO was added to the protein solution (1% v/v) in order to prevent signal stability problems during ITC measurements. A particular programmed titration involved a first false injection of 2 µl followed by each successive injection of 10 µl ligand at 260 seconds interval present in the syringe into the cell that contained protein. The stirring rate of the injector was kept at 320 rpm. The heat of dilution of ligand in buffer was subtracted from the titration data. The data was further analysed using MicroCal Origin 7.0 to calculate the stoichiometry of binding (*n*), enthalpy change (Δ*H*) and association constant (*K*_a_). These values were determined after the curve fitting of the binding isotherm to the ‘two-set of sites’ binding model software provided with the instrument.

### Cell proliferation study

MTT assay was done to determine the cytotoxic and antiproliferative properties of rutin and vanillin as described previously^3, 71^. Briefly, breast cancer cells (MCF-7), human cervical cancer cells (HeLa), human neuroblastoma (SH-SY5Y) cells and human embryonic kidney cells (HEK293T) were seeded in a 96 well plates at a concentration of 10 × 10^3^ viable cells per well. Afterwards, these cells were incubated with increasing concentration of rutin and vanillin (5 µM–200 µM). After 48 h of treatment, both medium and inhibitor were removed from the cells, washed twice with phosphate buffer saline (PBS) and then 20 µl MTT (from 5 mg/ml stock) and 100 µl DMEM was loaded to each well followed by 4–5 hours incubation in a CO_2_ incubator at 37 °C. Finally, the residual MTT medium was removed carefully and dissolves the crystals of formazan by adding 100 µl DMSO in each well. The micro-titer plates were then agitated for 15–20 minutes on an orbital plate shaker and then absorbance at 570 nm was determined on a titerplate reader (BioRad). The absorbance value so obtained was converted into percentage viability in comparison to the control cells (untreated cells/cells treated with media only). For cell proliferation studies doxorubicin has been taken as positive control.

### Cell apoptotic assay

Annexin-V staining was used to determine the cell apoptosis^72, 73^. MCF-7 cells were treated with rutin (80 µM) and vanillin (120 µM) (with IC_50_ dose, concentration at which cell viability decreases by 50%) for 48 h at 37 °C, and the control cells were treated with the media only. After treatment, nearly 2 × 10^6^ cells were trypsinized and washed two times with 5 ml of PBS by centrifuging at 1800 rpm for 4 min. FITC-Annexin-V staining was done by using FITC-Annexin-V kit according to the manufacturer’s instructions (BD-Biosciences, USA). 10,000 events for each sample were analyzed by flow cytometry BD FACS Canto and data analysis was performed with help of flowJo software.

### Reactive oxygen species determination by DCFDA and DHE staining

Total Reactive oxygen species determination (ROS) content inside the cells was determined using the DCFDA reagent. This assay measures various ROS such as H_2_O_2_ and hydroxyl radicals^74^. Approximately 70–80% confluent MCF-7 cells in 24-well culture plates were treated with increasing concentrations of rutin, vanillin (20–120 μM) and positive control H_2_O_2_, respectively and subsequently assayed for ROS levels estimation using the DCFDA fluorescent dye (Invitrogen Grand Island, NY). In brief, after the incubation time, cells were washed with 500 μl Kreb’s Ringer buffer (20 mM HEPES, 2 mM MgSO4, 10 mM dextrose, 127 mM NaCl, 1 mM CaCl2 and 5.5 mM KCl), prewarmed at 37 °C and then incubated at 37 °C in DMEM containing 10 μM DCFDA dye for 30 min. After that cells were washed twice with PBS, pH7.4, trypsinized and collected by centrifugation. Finally cells were resuspended in 300 μl of PBS and fluorescence was measured at Jasco spectroflourimeter (FP-6200) using a 5 mm quartz cuvette. The excitation and emission filters were set at 485/500–550 nm respectively. Similarly with some modifications, cytosolic superoxide levels were determined using dihydroxyethidium staining. Briefly, cells were grown on coverslips to 70–80% confluency and replaced the complete media with reduced serum media for overnight. After giving the treatments of rutin (IC_50_ = 80 μM) and vanillin (IC_50_ = 120 μM) for 4–5 hr, DHE was added at the final concentration of 10 μM, wrap the plate in aluminum foil and incubated at 37 °C for 30 min in dark. Following DHE staining, DAPI is used to satin nuclei of the cells as described earlier^71^. Fluorescence images were taken on Nikon-EclipseTS100 microscope.

### Statistical analysis

All the data are expressed as mean ± standard error from at least three independent experiments. Statistical analysis of data was performed using the Student t-test for unpaired samples and one-way ANOVA. Differences were considered significant at P < 0.05.

## Electronic supplementary material

Supplementary Figures
