# Supplementary material for: Elucidation of Dietary Polyphenolics as Potential Inhibitor of Microtubule Affinity Regulating Kinase 4: In silico and In vitro Studies
Source: Sci Rep. 2017 Aug 25;7:9470. doi: 10.1038/s41598-017-09941-4 (PMC5573368; doi:10.1038/s41598-017-09941-4)
Supplement: Supplementary file 1 — Supplementary Figures [file 41598_2017_9941_MOESM1_ESM.pdf]

Supplementary information

Elucidation of Dietary Polyphenolics as Potential Inhibitor of Microtubule Affinity Regulating Kinase 4: In silico and In vitro Studies

Parvez Khan[1], Shafikur Rahman[2], Aarfa Queen[1], Saaliqa Manzoor[1], Farha Naz[1], Gulam Mustafa Hasan[3], Suaib Luqman[4], Jihoe Kim[2], Asimul Islam[1], Faizan Ahmad[1] and Md. Imtaiyaz Hassan[1,*]

[1]*Center for Interdisciplinary Research in Basic Sciences, Jamia Millia Islamia, Jamia Nagar, New Delhi – 110025, India*

[2]*Department of Medical Biotechnology, Yeungnam University, Gyeongsan, 712-749, South Korea.*

[3]*Department of Biochemistry, College of Medicine, Prince Sattam Bin Abdulaziz University, Al-Kharj, Saudi Arabia.*

[4]*CSIR-Central Institute of Medicinal and Aromatic Plants, Lucknow, 226015, India*

Running Head: MARK4 inhibitors

***To whom correspondence should be addressed:**

Md. Imtaiyaz Hassan, Ph.D.

Assistant Professor

Centre for Interdisciplinary Research in Basic Sciences,
Jamia Millia Islamia,
New Delhi-110025,
E-mail: mihassan@jmi.ac.in

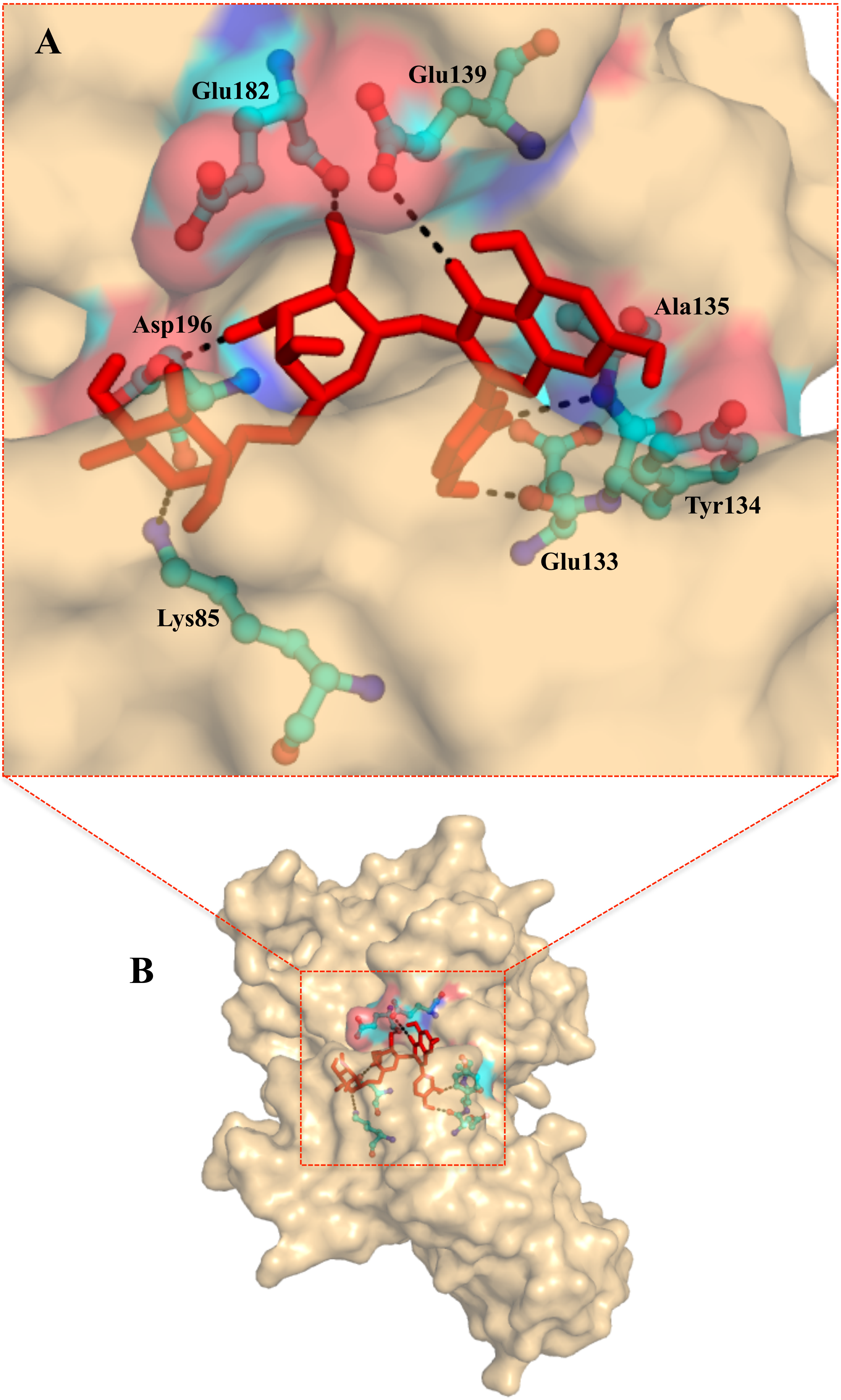

Figure S1: Binding of rutin with MARK4. **(A)** Enlarged surface view showing the binding of rutin with MARK4 **(B)** Surface view of rutin binding to MARK4. Interacting residues of MARK4 (ball and stick model) and rutin (stick model).

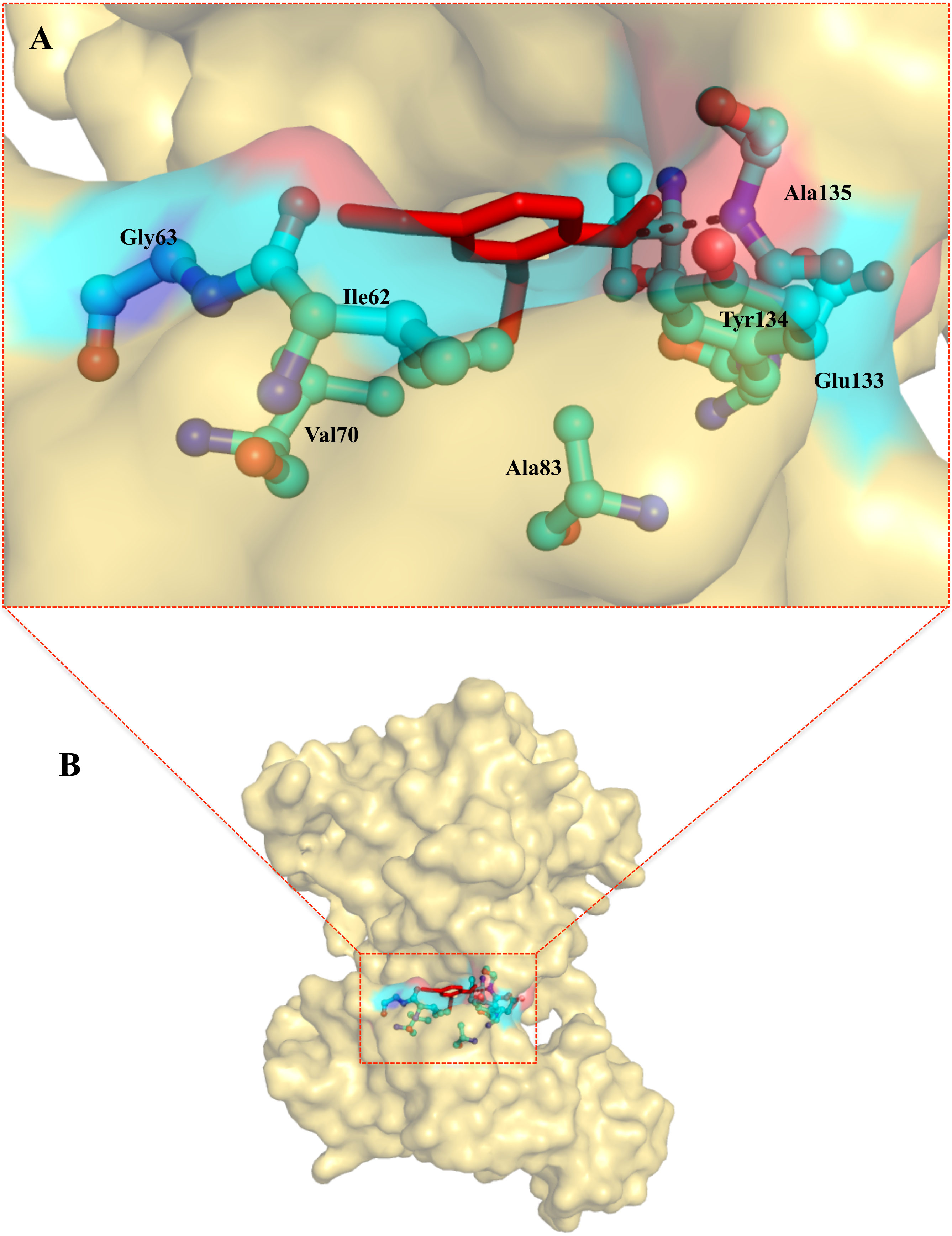

Figure S2: Binding of vanillin with MARK4. **(A)** Enlarged surface view showing the binding of vanillin with MARK4 **(B)** Surface view of vanillin binding to MARK4. Interacting residues of MARK4 (ball and stick model) and rutin (stick model).

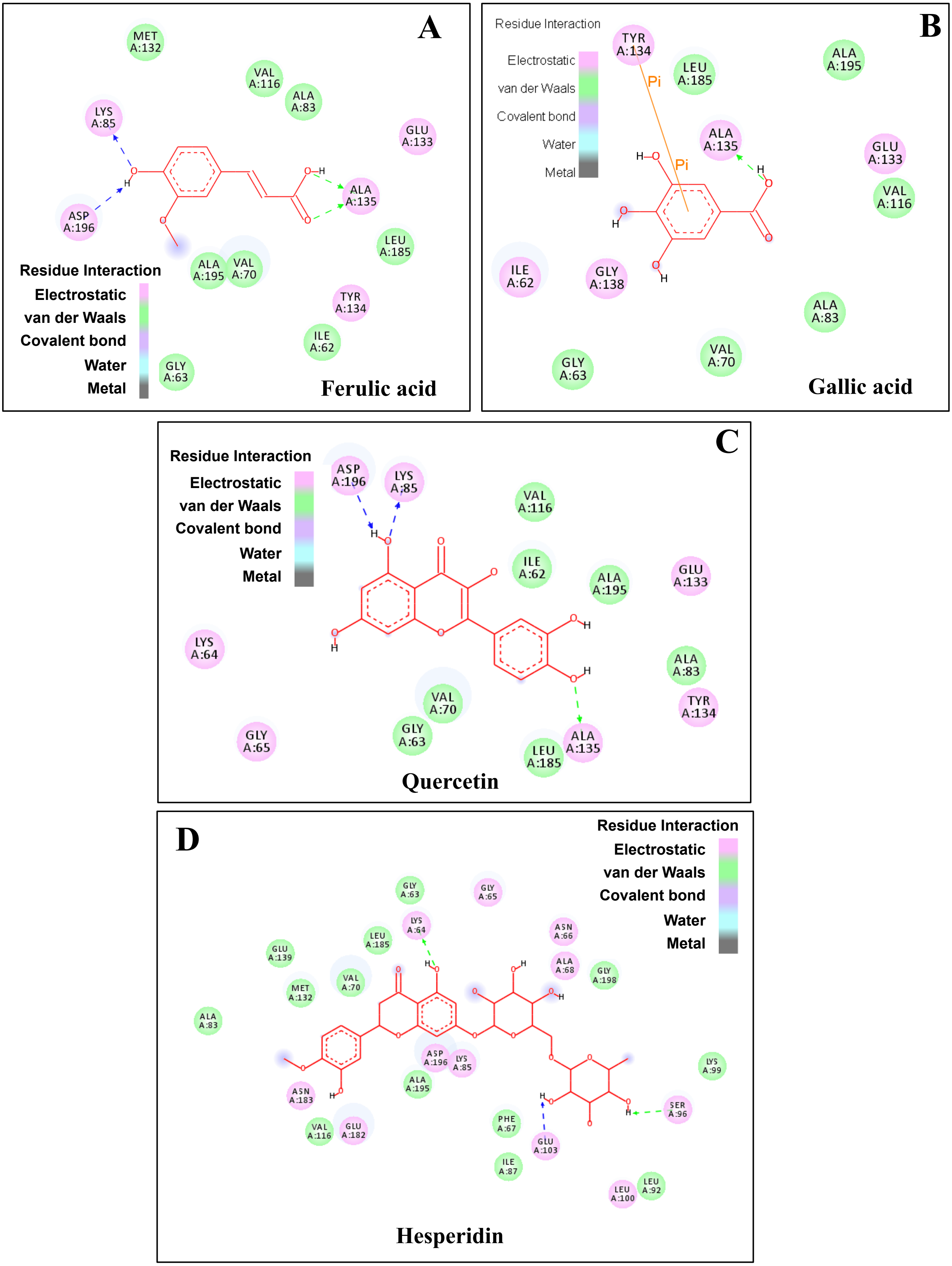

Figure S3: 2D schematic diagram of docking model of ferulic acid, gallic acid, quercetin and hesperidin with MARK4. Residues involved in hydrogen bonding, charge or polar interactions, Van der Waals interactions are represented by respective colour indicated in inset of figure.

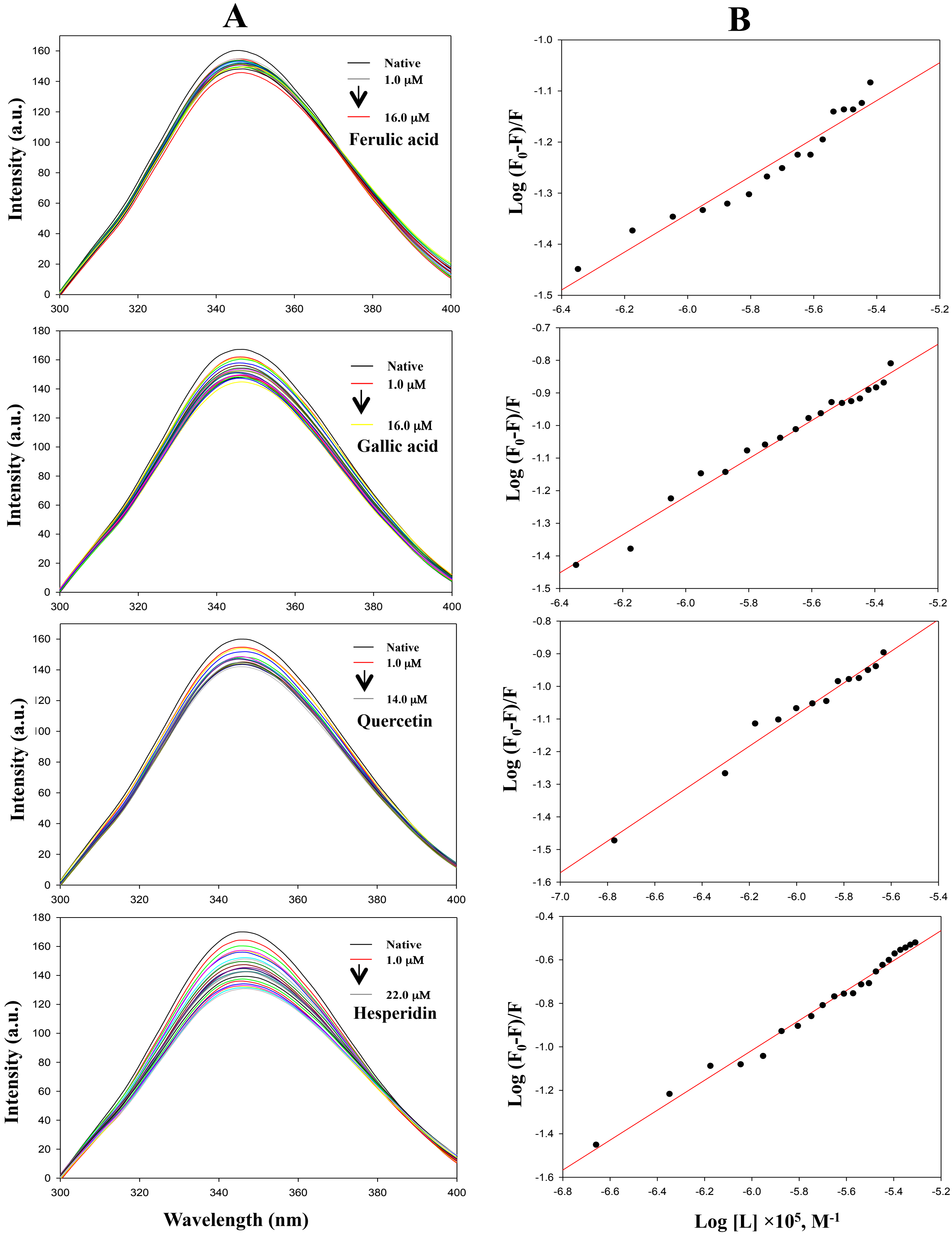

Figure S4. Binding pattern of ferulic acid, gallic acid, quercetin and hesperidin, respectively observed using fluorescence spectroscopy. **(A)** Fluorescence spectra of MARK4 (4µM) with increasing concentration of each compound (0-100µM). Excitation wavelength was fixed to 280nm and emission was recorded in the range 300-400 nm. **(B)** Modified Stern-Volmer plot showing quenching of MARK4 by each mentioned compound.

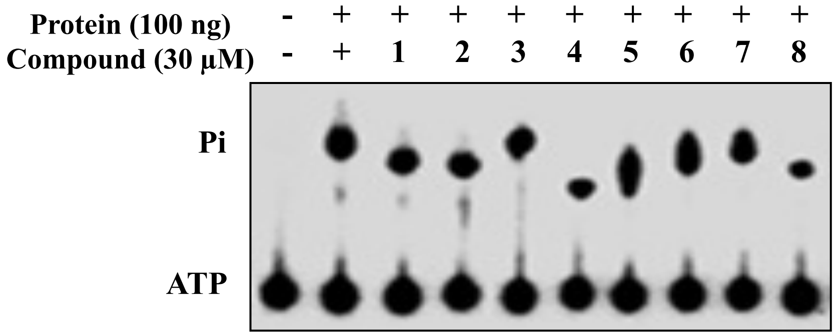

Figure S5: ATPase enzyme assay of MARK4. Shows the hydrolysis of Pi from ATP, position of Pi and ATP spots are indicated on left side. Lane 1, negative control (without protein); lane 2, 100 nM MARK4 (positive control); number 1&2= gallic acid, 3= quercetin, 4=rutin, 5= ferulic acid, 6&7= hesperidin and 8 represent the vanillin, respectively.
